# Dental care during pregnancy based on the pregnancy risk assessment monitoring system in Utah

**DOI:** 10.1186/s12903-019-0921-3

**Published:** 2019-11-06

**Authors:** Chandni Muralidharan, Ray M. Merrill

**Affiliations:** Department of Public Health, College of Life Sciences, Brigham Young University, 2063 Life Sciences Building, Provo, UT 84602 USA

**Keywords:** Dental, Health education, Insurance, Oral health, Pregnancy, PRAMS

## Abstract

**Background:**

Although receiving dental care is recommended for women during pregnancy, getting such care remains low. This study will identify the level of dental care received during pregnancy and factors associated with care for a group of pregnant women in Utah.

**Methods:**

Analyses were based on 2793 pregnant women completing the 2014–2015 Utah PRAMS survey. Descriptive and bivariate techniques were used.

**Results:**

Approximately 91.2% knew it was important to care for their teeth and gums during pregnancy, yet only 58.8% had their teeth cleaned during pregnancy. Those who knew such care was important were 1.4 (95% CI 1.1–2.0) times more likely to have their teeth cleaned during pregnancy. Although 18.8% needed to see a dentist for a problem, only 74.5% of them received treatment for the problem during pregnancy. Approximately 76.0% had dental insurance during pregnancy. Those with dental insurance were 1.9 (95% CI 1.5–2.4) times more likely to have their teeth cleaned and 1.6 (95% CI 1.2–2.2) times more likely to go to a dentist for needed treatment during pregnancy. Approximately 51.4% had a dental/health care worker talk with them about how to care for their teeth and gums. These women were more likely to know it was important to care for their teeth and gums during pregnancy (97.4% vs 87.6%, *p* < 0.0001). For women who received care for a problem during pregnancy, 70.0% had a dental/health care worker talk with them about how to care for their teeth and gums. These women were more likely to know it was important to care for their teeth and gums during pregnancy (95.2% vs 82.8%, *p* < 0.0001). Women who had their teeth cleaned the year prior to pregnancy were more likely to have their teeth cleaned during pregnancy (78.5% vs 21.5%, *p* < 0.0001).

**Conclusions:**

A large proportion of women knowing of the importance of dental care during pregnancy did not receive care. Knowledge of its importance during pregnancy, having a dental/health care worker talk with them about how to care for their teeth and gums, and having dental insurance during pregnancy are positively associated with dental care during pregnancy.

## Background

Poor dental hygiene and gum disease can increase the risk of heart attack, stroke, and adverse pregnancy outcomes [1–3]. During pregnancy, a woman’s body goes through several changes that can cause oral health problems, including hormonal changes, fluctuation in oral hygiene practices and eating patterns [4]. A 2012 national experts’ consensus statement concluded that dental care is both safe and effective throughout pregnancy, and that women should continue receiving treatment during their pregnancy [5]. Research supports that routine preventive, diagnostic, and restorative dental therapy, and periodontal treatment, among pregnant women does not increase the risk of adverse pregnancy outcomes [4]. Continued dental care during pregnancy is particularly important to avoid periodontal diseases [6–9] and subsequent risk of pre-eclampsia, preterm birth, and low birth weight infants [10–13].

Few studies have reported on the level of dental care received during pregnancy. However, those studies have shown lower levels of dental care during pregnancy. For example, in a study involving 2009–11 data from the Pregnancy Risk Assessment Monitoring System (PRAMS) in Hawaii, 50% had their teeth regularly cleaned before pregnancy compared with 33.4% during pregnancy [14]. In a study involving 1998 data for four states from the PRAMS, 23 to 35% of pregnant women went to the dentist during pregnancy, 12 to 25% suffered from an oral health problem, and, of those with a problem, only 45 to 55% received care [15]. The study further found that some women thought poor oral health during pregnancy was normal, believing that some types of dental care could harm their fetus. In a study involving 2004–08 data from PRAMS in Michigan, 26.0% reported that they needed dental care during their pregnancy, of which only 58.4% sought care [16]. Low levels of care may be because of concern among patients, dentists, and obstetricians regarding the safety of dental care during pregnancy. Lack of dental insurance may also play an important role. A better understanding is needed as to how these factors contribute to the use of dental care during pregnancy, according to specific subgroups of the population.

Research shows that some dentists and obstetricians contribute to the low use of dental care among pregnant women. In one study, only 40% of pregnant women were advised by their obstetric provider to seek dental care during pregnancy, and 10% were refused care during pregnancy by their dentist [17]. A recent survey of gynecologists found that they often mistakenly believed that dental x-rays (73%) and local anesthesia (59%) were unsafe for pregnant women [18].

Some healthcare workers are simply not counseling their patients about the importance of receiving dental care during pregnancy. In a study involving PRAMS data for 10 states in 2004–06, only 41% of pregnant women received oral health counseling [19]. A better understanding is needed of how such counseling impacts the use of dental care during pregnancy. Many dental and obstetrics workers may simply be unaware of the prenatal oral health guidelines, thereby causing them to not recommend dental care during pregnancy and to be less likely to talk with their patients about how to care for their teeth and gums during pregnancy. In a survey conducted in the United States of 60 dental school deans and 240 obstetrics and gynecology residency program directors 65% of deans and 45% of residencies were aware of prenatal oral health guidelines and 39% of residencies taught prenatal oral health [20]. In addition, it has been recommended that pregnant women should receive education on the importance of oral health care and safe medications during pregnancy in order to encourage home oral hygiene care [4, 5].

Not having insurance coverage can limit dental care during pregnancy [15, 16]. Pregnant women with either private or Medicaid insurance are less likely to receive dental care during pregnancy than their non-pregnant counterparts [21, 22]. The type of insurance has also been associated with use of dental care during pregnancy. In a PRAMS study, pregnant women with Medicaid insurance were 24 to 53% less likely to receive dental care than those covered by private insurance [15]. This is particularly concerning since Medicaid insurance for dental care during pregnancy, as it is available in most states, only provides coverage during their pregnancy and for 2 months postpartum [9], such that if dental care is delayed because of pregnancy, there may not be insurance to cover treatment later on.

Certain demographic characteristics and smoking and alcohol behaviors have been associated with failure to receive dental care during pregnancy. Research has shown that pregnant women are less likely to receive dental care during pregnancy if they are younger, a minority, not married, and have lower income and education [16, 23, 24]. Women who smoke cigarettes or drink alcohol also have lower use of dental care during pregnancy [24, 25].

From this previous research we can conclude that dental services are less frequently used among pregnant women, and that certain demographic groups, smokers, alcohol drinkers, and uninsured or publically insured pregnant women are least likely to use dental services. However, we know little about how knowledge of the importance of care during pregnancy and having a dental or health care worker talk with pregnant women about dental care influences whether dental care is received. In addition, we know little about the characteristics of pregnant women who are most likely to have dental insurance. The purpose of the current study is to identify the prevalence of dental services received (teeth cleaning and seeing a dentist for a dental problem) and factors related to receiving dental care (knowledge of the importance of care, having a dental or other health care worker talk with them about the importance of dental care, and insurance) among a group of women who completed the PRAMS survey in Utah. The impact of knowing the importance of receiving continued dental care during pregnancy, having a dental or other health care worker talk with them about such care, and insurance on actually receiving dental care during pregnancy will also be assessed.

## Methods

Analyses were based on data from the PRAMS 2014–2015 survey. PRAMS collects data on mother’s attitudes and experiences before, during, and shortly after pregnancy. Although PRAMS is conducted in many areas throughout the United States, this study focused on the Utah catchment area. Focus is given to pregnant women residing in Utah because of their relatively high fertility and birth rates and use of dental care. For example, from 2007 through 2016, fertility (births per 1000 women aged 15–44) and birth rates have been consistently higher in Utah than in other states in the country [26]. In 2016, the fertility rate was 76.2 and the birth rate was 16.5 per 1000 people in Utah [27]. Corresponding rates in the United States were 62.0 and 12.2, respectively [27]. In 2016, a high percentage of adults in Utah also visited the dentist or dental clinic within the last year for any reason—72.9% (69.5% of men and 76.3% of women) in Utah compared with 66.4% (64.1% of men and 69.7% of women) in the United States [28].

The study was reviewed by the IRB at Brigham Young University and given exempt status because PRAMS data are publicly available and subjects cannot be identified through linked identifiers.

### Pregnancy Risk Assessment Monitoring System

PRAMS is a population-based surveillance system that monitors maternal attitudes and experiences. The information is collected before, during, and shortly after pregnancy in mothers who delivered a live infant. The questionnaire covers an array of topics, including oral health. The sample consists of a random sample of pregnant mothers identified from birth certificates.

The sampling protocol was formulated by the Centers for Disease Control and Prevention and has a 60% response rate [29]. A detailed description of the data collection process has been provided elsewhere [30]. The primary data collection process for this study was a mixed-mode of mail and telephone, with personalized mailing packages, use of response incentives and rewards, and telephone follow-up for mail non-respondents. The telephone survey was conducted by a trained interviewer. Up to 5 contact attempts were made through the mail and up to 15 call attempts.

### PRAMS variables

Demographic measures included maternal age, race, ethnicity, marital status, annual household income, and years of education. Maternal cigarette smoking and alcohol drinking habits were also included in the study. The survey had a continuous numerical scale for maternal age, which was categorized as 15–20, 21–30, 31–40 and 41 and over. Maternal race was recoded as “white” and “other” because other represented different racial groups with small numbers, which may not be representative of the Utah population. There were 2234 whites, 479 other races, and 80 with missing race information. Maternal ethnicity was recoded as “Hispanic” and “non-Hispanic.” Marital Status was recoded as “married” and “other.” Annual household income was recoded into three categories: “$0–$26,000,” “$26,001–$52,000,” and “$52001 or more.” Years of maternal education had response options of “0–8 years,” “9–11 years,” “12 years,” “13–15 years,” and “≥ 16 years.” Smoked cigarettes in the past 2 years during, before, or after pregnancy and drank alcohol in the past 2 years during, before, or after pregnancy had response options of “yes” and “no.” Among the 394 who said “yes” to smoking in the past 2 years, some indicated they smoked in the last 3 months of pregnancy (i.e., 67 smoked 1–5 cigarettes, 43 smoked 6–10 cigarettes, 7 smoked 11–20 cigarettes, 3 smoked 21–40 cigarettes, and 3 smoked 41 or more cigarettes). Among 934 who said “yes” to alcohol drinking in the past 2 years, a small number said they consumed alcohol in the last 3 months of pregnancy (i.e., 1 said 4–6 drinks a week and 8 said 1–3 drinks per week). Because of small numbers we only included in the study whether or not they smoked cigarettes or drank alcohol in the past 2 years.

Questions related to dental services during the women’s most recent pregnancy were: “I knew it was important to care for my teeth and gums during my pregnancy,” “A dental or other health care worker talked with me about how to care for my teeth and gums,” “I had my teeth cleaned by a dentist or dental hygienist,” “I had insurance to cover dental care during my pregnancy,” “I need to see a dentist for a problem,” and “I went to a dentist or dental clinic about a problem.” Response options for these items were “yes” and “no.” An additional question was asked to determine whether teeth cleaning occurred the year prior to getting pregnant, as follows: “I had my teeth cleaned by a dentist or dental hygienist any time during 12 months before my pregnancy with my new baby.” Response options for this item were “yes” and “no.”

Sampling weights were applied to the data in order to account for over- or under-sampling, thereby providing a representative group of all pregnant women in Utah.

### Statistical techniques

Counts and percentages were used to describe the data. Bivariate analyses were evaluated for statistical significance using the Rao-Schott chi-square test. Relative risks were obtained using the Mantel-Haenszel method. Unadjusted and adjusted relative risks were presented, with adjustment made for maternal age, race, ethnicity, marital status, annual household income, education, cigarette smoking, and alcohol drinking status. Two-sided tests of significance were based on the 0.05 level against a null hypothesis of no association. Data was evaluated using the statistical software package PC-SAS (version 9.4; SAS Institute, Inc., 2014).

## Results

**Fig. 1:**
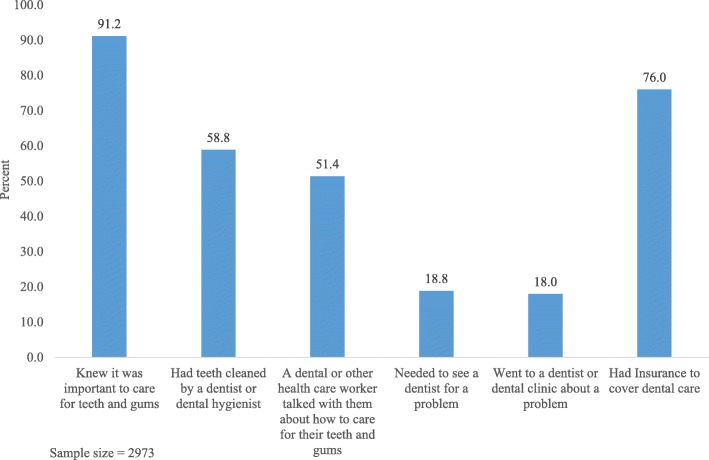
Agreement with selected items related to dental care during the women’s most recent pregnancy

A description of the 2793 study participants is presented according to selected demographic, smoking, and alcohol variables in Table 1. Most women are in the age range 21–30 years, white, non-Hispanic, married, have an annual household income of $52,000 or more, have more than a high school education, do not smoke cigarettes, and do not drink alcohol. The table also presents bivariate analyses between the demographic, smoking, and alcohol variables and responses to selected items that relate to dental care during the subjects most recent pregnancy. The percent of women responding “yes” to each of these selected items is presented in Fig. 1.

**Table 1:** Description of study participants and bivariate analyses, assessing the association between responses to dental care related items during the most recent pregnancy and selected demographic, smoking, and alcohol variables

Demographic, smoking, and alcohol variables			Knew it was important to care for teeth and gums		Had teeth cleaned by dentist or dental hygienist during pregnancy		A dental or other health care worker talked with them about how to care for their teeth and gums		Needed to see a dentist for a problem		Went to see a dentist for a problem during pregnancy		Had insurance to cover dental care	
Maternal age	No.	%	% Yes	Pr > ChiSq	% Yes	Pr > ChiSq	% Yes	Pr > ChiSq	% Yes	Pr > ChiSq	% Yes	Pr > ChiSq	% Yes	Pr > ChiSq
15–20	261	6.2	82.6	0.0007	53.8	0.0028	50.4	0.9163	22.9	0.1748	23.5	0.0200	74.2	0.6123
21–30	1657	61.3	92.0		56.6		51.7		19.4		19.0		76.7	
31–46	875	32.5	91.1		63.9		50.9		17.0		15.1		75.0	
Maternal race														
White	2234	86.3	93.1	< 0.0001	61.0	< 0.0001	52.2	0.0683	19.0	0.6716	17.9	0.8689	78.1	< 0.0001
Other	479	13.7	78.7		45.4		46.3		18.0		18.3		62.5	
Missing	80													
Maternal ethnicity														
Hispanic	579	15.6	81.5	< 0.0001	44.2	< 0.0001	44.8	0.0114	19.1	0.9978	17.2	0.6281	56.1	< 0.0001
Non-Hispanic	2095	84.4	92.8		61.5		52.4		19.09		18.4		79.6	
Missing	119													
Marital status														
Married	2150	82.9	92.4	< 0.0001	61.29	< 0.0001	52.1	0.1937	17.3	< 0.0001	16.1	< 0.0001	77.2	0.0073
Other	634	17.1	84.8		47.4		48.2		26.9		27.1		70.5	
Missing	9													
Annual household income														
$0–$26,000	946	29.4	86.7	< 0.0001	45.5	< 0.0001	46.0	0.0040	24.4	0.0002	23.3	0.0008	71.3	< 0.0001
$26,001–$52,000	725	27.7	92.5		54.9		51.6		18.8		16.0		73.6	
$52,001 or More	925	42.9	93.8		71.0		55.5		15.2		15.5		82.1	
Missing	197													
Maternal education (years)														
0–8	84	1.5	72.0	< 0.0001	27.4	< 0.0001	33.3	0.0207	21.6	< 0.0001	9.4	< 0.0001	17.9	< 0.0001
9–11	452	8	82.0		43.1		45.9		27.9		23.8		63.0	
12	770	19.3	89.0		53.5		49.5		23.1		22.8		74.7	
13–15	674	35.6	93.5		57.1		51.8		20.9		20.1		79.8	
≥ 16	669	35.6	93.4		68.8		54.3		12.3		12.2		79.1	
Missing	144													
Smoked cigarettes in the past 2 years during, before, or after pregnancy														
Yes	396	11.2	88.3	0.0915	51.4	0.0145	47.1	0.1641	29.3	< 0.0001	29.2	< 0.0001	82.7	0.0091
No	2355	88.8	91.5		59.0		51.9		17.6		17.6		75.2	
Missing	42													
Drank alcohol in the past 2 years during, before, or after pregnancy														
Yes	945	31.7	90.8	0.534	55.6	0.0471	48.2	0.0618	23.1	0.0012	21.2	0.0130	80.7	0.0009
No	1807	68.3	91.6		60.5		52.9		16.9		16.4		73.9	
Missing	41													

Notes: Percentages are weighted. Only 27 women were older than age 40 years

As shown in the table, women who knew it was important to care for their teeth and gums during pregnancy were significantly more likely to be at least 21 years of age, white, non-Hispanic, married, and have higher income and education. Women who had their teeth cleaned by a dentist or dental hygienist during pregnancy were significantly more likely to be older, white, non-Hispanic, married, non-cigarette smokers, non-alcohol drinkers, and have higher income and education. Women who had a dental or other health care worker talk with them about how to care for their teeth and gums during pregnancy were significantly more likely to be non-Hispanic and have higher income and education. Women that had to see a dentist for a problem were significantly more likely to not be married, smoke cigarettes, drink alcohol, and have lower income and lower education. Women who went to a dentist for a problem during pregnancy were significantly more likely to be younger, not married, smoke cigarettes, drink alcohol, and have lower income and education. Finally, women that had dental insurance were significantly more likely to be white, non-Hispanic, married, smoke cigarettes, drink alcohol, and have higher income and education.

A higher percent of women who knew it was important to care for their teeth and gums during pregnancy had their teeth cleaned during pregnancy (61.3% vs. 33.6%, *p* < 0.0001); women who knew it was important to care for their teeth and gums during pregnancy were 1.8 (95% CI 1.3–2.5) times more likely to have their teeth cleaned during pregnancy. The ratio became 1.4 (95% CI 1.1–2.0) after adjusting for the variables in Table 1. A higher percent of women having dental insurance had their teeth cleaned during pregnancy (67.4% vs 31.7%, *p* < 0.0001), with unadjusted and adjusted ratios of 2.1 (95% CI 1.7–2.6) and 1.9 (95% CI 1.5–2.4), respectively. Of those who said they needed to see a dentist for a problem during pregnancy, 74.5% went to the dentist for care. A higher percent of women having dental insurance received care for a problem during pregnancy (20.2% vs 10.6%, *p* < 0.0001), with unadjusted and adjusted ratios of 1.9 (95% CI 1.4–2.6) and 1.6 (95% CI 1.2–2.2), respectively.

Among those women who had their teeth cleaned during pregnancy, 75.2% had a dental or health care worker talk with them about how to care for their teeth and gums. Among those who had this talk, 97.4% agreed that they knew it was important to care for teeth and gums during pregnancy, whereas among those who did not have this talk, 87.6% agreed that they knew it was important to care for teeth and gums during pregnancy (*p* < 0.0001). Among those who went to a dentist or dental clinic about a problem, 70.0% had a dental or health care worker talk with them about how to care for their teeth and gums. Among those who had this talk, 95.2% agreed that they knew it was important to care for teeth and gums during pregnancy, whereas among those who did not have this talk, 82.8% agreed that they knew it was important to care for teeth and gums during pregnancy (*p* < 0.0001).

Dental cleaning by a dentist or dental hygienist during pregnancy was much more common if the patient received a cleaning the previous year (Table 2). Most women were in this category. The second most common group did not receive a cleaning during pregnancy or in the year before. Of women with a dental cleaning the year prior to pregnancy, 77.3% had their teeth cleaned during pregnancy. Of women without a dental cleaning the year prior to pregnancy, only 21.0% had their teeth cleaned during pregnancy. Dental insurance was significantly associated with receiving a cleaning during pregnancy.

**Table 2:** Distribution of dental cleaning by a dentist or dental hygienist the year prior to and during pregnancy by insurance status during pregnancy

Dental cleaning by a dentist or dental hygienist				Dental insurance during pregnancy	
Previous year	During pregnancy	No.	%	% Yes	Pr > ChiSq
Yes	Yes	1251	51.1	86.2	< 0.0001
Yes	No	368	14.0	69.4	
No	Yes	231	7.7	92.9	
No	No	869	27.2	55.5	

Percentages are weighted

## Discussion

This study focused on pregnant women in Utah who completed the PRAMS survey. Although 91.2% knew it was important to care for their teeth and gums during pregnancy, only 58.8% had their teeth cleaned during this time. However, this percentage is higher than observed in other studies [14–16]. It may be higher because women in the current study had greater annual household income and education, both of which were seen to more positively correlate with teeth cleaning than in other studies.

Of those who indicated that they needed to see a dentist for a problem during pregnancy, only 74.5% received such care. However, this percentage is higher than in other studies. In contrast, approximately half in one study and 58.4% in another study who reported having dental problems went to a dentist or dental clinic for care [15, 16]. Higher annual household income, education, and insurance to cover dental care during pregnancy may explain the higher levels of service received in our study.

The results showed that women most prone to not receive dental care during pregnancy were younger, minorities, not married, and had lower income and education. Other studies have also associated these characteristics with lower oral care during pregnancy [16, 23, 24]. With the exception of maternal age, these other characteristics were also associated with lower levels of insurance to cover dental care during pregnancy. Health policy measures aimed to improve insurance for dental care during pregnancy should focus on these vulnerable groups.

A fear that dental care can be dangerous to the unborn child has been observed in previous studies as a primary deterrent to dental care during pregnancy [9, 15, 31]. This misconception is also shared with some dentists and obstetric providers [17]. One study found that many dental school deans and obstetric and gynecology residency program directors are not aware of oral health guidelines for pregnant women [20]. Improving awareness of the guidelines is an important step, but also movement is needed toward providing increased education, targeted to specific high-risk populations, to alter the misconception that dental care during pregnancy is unsafe. It has been recommended that pregnant women should receive education from dentists or health care workers about the importance of oral health care and safe medications during pregnancy to improve use of dental care at this critical time [4, 5]. Research has shown that oral health education can effectively improve the knowledge, attitude, and practice of oral health [32]. The importance of dentists and health care workers educating pregnant women about dental care during pregnancy is identified in the current study wherein a higher percent of women who knew it was important to care for their teeth and gums during pregnancy had their teeth cleaned by a dentist or dental hygienist during pregnancy. The results also showed a positive association between a dental or other health care worker talking with them about how to care for their teeth and gums and knowing it was important to care for their teeth and gums during pregnancy.

Having insurance to cover dental care during pregnancy was associated with greater use of teeth cleaning and going to a dentist or dental clinic to receive treatment for a problem. Other research has shown that not having insurance limits obtaining dental care during pregnancy [15, 21, 22]. A national survey of 801 pregnant women and new mothers (within the past 12 months) between ages 21 and 45 years conducted by Cigna found that pregnant women were twice as likely to skip a dental checkup if they did not have insurance coverage [33]. In our study, dental insurance was more common among white, non-Hispanic, married, higher income, and more educated women. We did not find an association between age and having dental insurance during pregnancy, as found in another study where older pregnant women were more likely to be insured [23]. We found that dental insurance during pregnancy was positively associated with teeth cleaning and going to a dentist or dental clinic to receive treatment for a problem. Thus, the need is emphasized for possible financial assistance of dental care among pregnant women, particularly among the at-risk groups identified.

Those more likely to need to see a dentist because of an oral health problem were older, not married, had lower income, had fewer years of education, smoked cigarettes, and drank alcohol. As we observed, non-married women may be less likely to receive care for needed dental problems because they are less likely to have insurance. Other research has shown cigarette smoking and lower income to be associated with failure to receive dental care during pregnancy [24, 25]. Regarding alcohol, its use has an effect on systemic, physiologic, and mental health, which thereby affects oral health [25]. Alcoholics may not be in a conscious and coherent state of mind to have a good relationship with their health care professional. Their altered mental state may impair their ability to understand oral health education/instructions, resulting in disregard for oral health practices or dental neglect. Additionally, alcohol causes dehydration, increasing the likelihood of developing dry mouth, which is conducive to a cariogenic environment. Tobacco also causes exacerbation or suppression of the immune system, thus inhibiting the defense mechanisms of the oral health cells [25]. Race/ethnicity were not significantly associated with needing to see a dentist for an oral health problem. This result is inconsistent with the Centers for Disease Control and Prevention (CDC) report that Non-Hispanic blacks, Hispanics and Alaskans generally have poor oral health compared with other racial groups [34]. Small numbers of non-whites and Hispanics in Utah may have influenced our findings.

Having teeth cleaned by a dentist or dental hygienist during pregnancy was much more common among women who had their teeth cleaned the previous year. Approximately 51.1% of women had their teeth cleaned during pregnancy in the previous year, but 27.2% did not have their teeth cleaned during pregnancy or in the previous year. This latter group was the least likely to have dental insurance. Research has recommended women to see a dentist both before and during pregnancy [9]. Establishing a habit of visiting the dentist regularly prior to pregnancy will not only improve the oral health of the mother but indirectly affect the future oral health of the child [9].

The results of the current study may contribute to improving dental care during pregnancy by both identifying the need for improvements in dental care and establishing factors associated with receiving this care. Dental care during pregnancy can improve by better educating women about the need for such care and assisting them in obtaining insurance during pregnancy. More vulnerable groups who could benefit most from patient intervention have been identified in this study. Further research may explore specific ways to effectively educate obstetric providers and dentists, as well as patients, about the importance of providing dental care during pregnancy. Whether better provider knowledge equates to being more likely to educate their patients, and how effective this communication is at improving patient dental care during pregnancy, deserves further consideration. Research may also explore effective ways to better educate patients with lower income and education, and of minority status. Finally, previous research has shown women with Medicaid as being less likely to receive dental care [15]. Reasons for this outcome need to be better understood.

There are currently certain educational efforts in place aimed at improving patient use of dental care during pregnancy. The Health Resources and Services Administration (HRSA) of the U.S. Department of Health and Human Services, recently released Integration of Oral Health and Primary Care Practice [35], which is an outline of inter-professional core clinical competencies for oral health, to be implemented by primary care providers. Smiles for Life: A National Oral Health Curriculum, contains a course specifically aimed at the oral health of pregnant patients [36]. These and other resources about oral health and corresponding inter-professional competencies for women’s health care providers and students are presented elsewhere [37].

The study was limited in that the PRAMS survey is cross-sectional, which, with a 60% response rate, may be prone to response bias. Chance findings are possible, but unlikely because of the large sample size, with the possible exception for certain racial/minority groups. For this reason we combined certain racial and ethnic groups. Finally, in order to determine a relationship between teeth cleaning before pregnancy and during pregnancy, we asked questions related to during pregnancy and the 12 months prior to pregnancy. However, not all women know exactly when they get pregnant. It is possible for some to be several days or weeks into their pregnancy without knowing they are pregnant.

## Conclusion

Previous research has led experts to conclude that dental care during pregnancy is both safe and effective at reducing the risk of adverse personal health and pregnancy outcomes. Current guidelines and resources are in place and educational efforts underway nationally to increase patient use of dental services during pregnancy. A description of those women who are least likely to receive dental care during pregnancy, as provided by this study, may be useful in further focusing the guidelines, resources, and educational efforts. Since women who knew it was important to care for their teeth and gums during pregnancy were much more likely to receive dental care during pregnancy, it is evident that efforts to educate pregnant women of the benefits of continued dental care through pregnancy is needed. However, this requires educating dentists and obstetricians of the guidelines and recommendations for dental care for pregnant women. In addition, the strong association between having dental insurance and receiving dental services during pregnancy emphasizes the need for health policy to improve accessibility of dental insurance among vulnerable populations. The current study identifies the characteristics of women least likely to receive dental care during pregnancy.

## Data Availability

Data were provided by the Utah Pregnancy Risk Assessment and Monitoring System (PRAMS), a project of the Utah Department of Health (UDOH), the Office of Vital Records and Health Statistics of the UDOH, and the Centers for Disease Control Prevention and Prevention (CDC) of the U.S. Health and Human Services Department. This report does not represent the official views of the CDC or of the Utah Department of Health.

## Abbreviations

CDC: Centers for Disease Control and Prevention
HRSA: Health Resources and Services Administration
PRAMS: Pregnancy Risk Assessment Monitoring System
UDOH: Utah Department of Health
